# Evaluation of a training for managers in using active and personal communication to stimulate employee participation in a workplace smoking cessation program

**DOI:** 10.18332/tpc/208807

**Published:** 2025-08-29

**Authors:** Floor A. van den Brand, Julia M. van Koeveringe, Onno C. P. van Schayck, Gera E. Nagelhout

**Affiliations:** 1 Department of Family Medicine, Care and Public Health Research Institute, Maastricht University, Maastricht, The Netherlands; 2 Department of Surgery, Radboudumc, Nijmegen, The Netherlands; 3 Department of Health Promotion, Care and Public Health Research Institute, Maastricht University, Maastricht, The Netherlands; 4 Centre of Expertise Perspective in Health, Avans University of Applied Sciences, Breda, The Netherlands

**Keywords:** smoking cessation, low socioeconomic position, workplace health promotion, communication training

## Abstract

**INTRODUCTION:**

Workplace smoking cessation programs are effective in reducing smoking prevalence among employees with a lower socioeconomic position (SEP). However, effective participation of these employees remains challenging. This study evaluates a communication training program designed to equip managers with the skills to personally and actively engage employees in workplace smoking cessation initiatives.

**METHODS:**

This study, conducted in the Netherlands (September 2021–March 2023), used Intervention Mapping to develop a 90-minute interactive webinar to improve managers’ knowledge, attitudes, self-efficacy, and communication skills related to talking with employees about smoking cessation. A total of 107 managers participated. A mixed-methods approach was used for evaluation, including pre- and post-webinar questionnaires (completed by 50 participants) and two rounds of semi-structured interviews with 18 managers, around 3 and 12 months post-training.

**RESULTS:**

The training led to a small increase in self-efficacy regarding smoking cessation conversations from 3.2 to 3.5 on a scale of 1 to 5 (p=0.015). In the first round of interviews, 5 of the 18 managers had engaged in personal conversations with employees about smoking cessation. Facilitators were a strong belief in one's own ability to succeed; opportunities to practice communication skills; positive past experiences addressing smoking behavior; supportive company regulations and national smoke-free policies and assistance provided by colleagues. Barriers included low confidence in communication skills; perceived time burden; limited motivation or perceived role responsibility; the social sensitivity of the topic and fear of negative reactions; the absence of an in-house cessation program and insufficient organizational support. One year post-training, only one manager had consistently implemented personal communication strategies, while most others cited unchanged barriers and a lack of organizational support as reasons for inaction.

**CONCLUSIONS:**

While managers recognized the importance of personal communication for smoking cessation, implementation and maintenance were limited due to personal and organizational barriers. Future training programs should be more extensive and ensure structural organizational support for workplace smoking cessation initiatives.

## INTRODUCTION

Workplace health promotion programs can be effective in promoting health behaviors among adults, and have been shown to be effective in promoting smoking cessation^1-3^. Tobacco smoking is more prevalent among lower socioeconomic groups and is an important contributor to health disparities^4^. Workplace smoking cessation programs have been shown to be effective among people with a lower socioeconomic position (SEP) and have the potential to reduce these socioeconomic differences in health^5,6^. However, in order to effectively reduce the gap in smoking prevalence, it is essential that people with a lower SEP are effectively reached to participate in workplace smoking cessation programs. Achieving high rates of participation in workplace health promotion programs remains a challenge^3,7,8^. Most research on workplace smoking cessation programs has focused on the effectiveness of smoking cessation programs in general^9,10^, and little is known about how to effectively reach employees with a lower SEP to participate in such a program.

In 2018, the Dutch national government and more than seventy civil society organizations signed the National Prevention Agreement, committing to a smoke-free generation by 2040. As part of this agreement, companies are encouraged (though not legally required) to implement smoke-free policies for their employees, buildings, and premises, including offering support for smoking cessation. Yet smoking remains prevalent: in 2023, 24.4 % of lower educated adults and 24.8 % of those in the lowest income group still smoked, compared with 13.5 % and 11.3 % in their higher SEP counterparts^11,12^. These persistent disparities highlight the need for strategies that effectively engage lower SEP employees in workplace cessation programs.

To investigate how to encourage participation in workplace smoking cessation programs among people with a lower SEP, we previously conducted qualitative interview studies in the Netherlands with both employers and employees who smoked^13,14^. Both indicated that passive communication methods such as posters, e-mail or intranet messages, were not effective in reaching employees with a lower SEP and were not sufficient to encourage them to participate in a smoking cessation program. Instead, they recommended an approach in which managers approach employees personally to talk about the smoking cessation program. However, employers also felt that they lacked the communication skills to talk about smoking cessation, and employees stressed that it was important for employers not to use a judgmental tone^13,14^. Similarly, a previous study found that employers lacked the necessary skills to effectively communicate with employees about lifestyle changes and motivate them to participate in workplace health promotion programs. As a result, it was suggested by employers that supervisors should receive training on how to promote a healthy lifestyle for their employees ^15^.

To address these barriers in communicating about health behavior, we developed a training program for employers with the aim to encourage them to actively and personally communicate with their employees about a workplace smoking cessation program. In the current study, we aim to evaluate the communication training. We specifically aim to: 1) evaluate whether the communication training influenced employers’ knowledge, attitudes, outcome expectations and self-efficacy regarding personal communication; 2) identify the barriers and facilitators for communicating with employees about smoking cessation; and 3) evaluate whether employers who participated in the communication training implemented and maintained personal communication strategies since the training.

## METHODS

### Development of the communication training

Between January and September 2021, we used Intervention Mapping^16,17^ to develop a communication training program in co-creation with various experts. Our expert panel comprised 19 individuals (6 men and 13 women), all of whom were born and educated in the Netherlands. The panel included people who smoke, researchers specialized in health promotion and smoking cessation, smoking cessation coaches, communication experts, experts in health promotion among lower socioeconomic groups, an addiction physician and an occupational health physician. As part of the Intervention Mapping steps, we created a Logic Model of Change specifying change or the determinants most relevant to managers’ behavior^17^. The selected determinants included attitudes, skills and self-efficacy, and normative beliefs (Theory of Planned Behavior); knowledge and outcome expectations (Social Cognitive Theory) and perceived barriers (Health Belief Model)^18-20^. Prospective participants completed an online registration form in which they listed what they hoped to learn (Supplementary file Material 3). These needs were mapped onto the change objectives and used to finetune session content, ensuring the final training directly addressed managers’ stated questions and preferences. In order to develop guidance on how to communicate with employees who smoke in a non-judgmental and helpful way, and to formulate example sentences, additional individual sessions were held with a person who smokes, two smoking cessation coaches (one of whom used to smoke), and an addiction physician with specific expertise in smoking cessation.

### Delivery of the communication training

The communication training that was developed was delivered in the form of a live interactive webinar of 90 minutes. Subscription to the communication training was free from costs. The webinar was recorded and made available on YouTube and on the project website for viewing on demand afterward^21^. The first part of the communication training consisted of two short presentations from scientific experts on socioeconomic differences in smoking and the importance of personal communication for reaching employees with a lower SEP, and included a video of a company manager who shared his successful approach for stimulating employees to enroll in a workplace smoking cessation program, consisting of an active and personal approach, creating a bond with employees and a feeling that support is available if they need it. The second part of the communication training consisted of two actors who performed an interactive role-play between a manager and an employee who smoked. During the role-play, participants were asked to provide suggestions on how the actor-manager should approach the conversation. This part also included breakout rooms with 4 people each, where participants could exchange ideas and share their experiences. In the last part of the communication training, an addiction physician and the employeeactor provided a step-by-step plan on how participants could effectively talk with employees about smoking cessation. After the session, every participant received a digital factsheet that distilled the key takeaways, included a stepbystep conversation guide with sample phrases, and contained direct links to relevant websites for further information.

### Design

The current study was conducted in the Netherlands between September 2021 and March 2023. The training was provided on 14 September and 16 November 2021. Recruitment for the training was conducted through open promotion channels. The training was promoted on social media and through digital newsletters of our research organizations, health funds and trade organizations. The training was promoted as a free 1.5-hour online webinar aimed specifically at human resource managers, personnel and organization (P&O) professionals, managers, business owners, and team leaders. The promotional materials emphasized that the webinar would be interactive and teach participants how to communicate in a personal and supportive way with employees about smoking cessation. Individuals interested in the topic were able to register via an online sign-up form, resulting in a self-selected sample of participants. No additional selection criteria were applied. Short online questionnaires were distributed to every participant before and after the communication training. In addition, we conducted two rounds of semi-structured qualitative interviews with 18 employers who had attended the communication training.

### Questionnaires

Upon registration for the communication training, we asked people about their occupation and the sector and size of the company they worked at. In order to measure self-efficacy for personal communication about smoking cessation, we used the following statements of which the responses were averaged into a single score (Pearson’s r (48)=0.53, p<0.01):

*‘I am convinced that in the next three months I could personally address employees who smoke about quitting smoking if I wanted to’* and

*‘I think it is easy to personally address employees who smoke about quitting smoking in the next three months’*.

Both items could be answered on a 5-point Likert-scale ranging from 1 (completely disagree) to 5 (completely agree). Directly after the communication training, self-efficacy was measured again using the same items and intention to personally communicate with employees who smoke was measured with the following statement:

*‘I am committed to personally address employees who smoke about quitting in the next three months’*

which was measured on the same Likert scale. Differences in self-efficacy scores between pre- and post-measurement were assessed in SPSS using a paired-samples t-test.

### Recruitment

Participants in the communication training who had provided their consent to be approached for an interview were invited by telephone and email. We conducted individual qualitative interviews with persons in managerial positions in organizations in the Netherlands that participated in the communication training. A first round of 18 interviews was conducted on average three (range: 1–5) months after the communication training between November 2021 and March 2022. The interviews in the first round lasted between 20 and 60 minutes. About a year after the initial interviews, between October 2022 and March 2023, the same interviewees were contacted again for a shorter second interview. Of the 18 interviewees, nine agreed to participate in a second interview, and three agreed to answer a few questions on the telephone without audio recording, because they felt they had no additional information to report since the last interview. Three individuals could not be reached because they had switched jobs, with one individual we were not able to schedule an interview, and two interviewees were not approached for a second interview because the first interview revealed that within their type of job, they were unable to communicate with employees about smoking. The recorded interviews lasted between 15 and 35 minutes. Participants gave their written consent for participating in the interviews. The interviewees received €20 as compensation for their time.

### Data collection and outcomes

The interviews were in Dutch and conducted online over Zoom by FvdB, CIHB and JvK. FvdB and CIHB were experienced interviewers, FvdB trained JvK in interviewing. A semi-structured interview guide (Supplementary file Material 1) was used for the interviews. The main aim of the interviews was to assess whether participants had implemented personal communication about smoking cessation in their organization after the communication training and to identify remaining barriers and facilitators. In addition, we asked about reasons to participate in the communication training, what participants learned from the communication training, whether they disseminated the information from the communication training within their organization, and we inquired about the influence of the communication training on psychosocial determinants including knowledge, attitude, intention, and outcome expectations regarding personal communication with employees about smoking cessation. In the second round of interviews, we asked whether managers in the past year had personally communicated with their employees about smoking cessation and asked about barriers and facilitators and (changes in) psychosocial determinants.

### Analysis

The interviews were audio recorded, transcribed verbatim and uploaded to NVivo12 for coding and analyses. The interviews were coded through thematic content analysis. FAB, CIHB and JK double coded transcripts. All coders had backgrounds in health promotion and were aware of the potential influence of their perspectives on data collection and interpretation. Both an inductive and deductive approach was used to analyze the transcript and generate codes. Discrepancies were discussed in regular meetings until consensus was reached. Codes were combined and rearranged into themes and subthemes, until a final thematic framework was agreed upon. CIHB and FAB then created a matrix in which responses were summarized and added to the matrix. In the final stage of interpretation of the results, FAB and JK examined the matrix for connections across themes.

## RESULTS

### Quantitative results

In total, 107 people participated in the communication training, of which 50 completed both a pre- and postwebinar questionnaire. The mean self-efficacy score was slightly higher after the training than before (3.2 and 3.5, respectively; p=0.015). The respondents’ intention to personally address employees who smoke about quitting in the next three months was 3.5.

### Qualitative results

#### Interviewed participants

We interviewed 11 women and 7 men. The majority of the participants was aged ≥50 years and had a moderate or high level of education. Most were employed in human resources or (occupational) health (Table 1). The analysis revealed no meaningful variation in thematic coding of interview data between male and female participants. Therefore, results are presented for the full sample.

**Table 1:** Characteristics of interview participants (N=18)

*Characteristics*	*Categories*	*n*	*%*
**Gender**	Men	7	38.9
	Women	11	61.1
**Age** (years)	>30	1	5.6
	30–39	3	16.7
	40–49	1	5.6
	50–59	9	50.0
	≥60	4	22.2
**Education level^†^**	Low	3	16.7
	Moderate	8	44.4
	High	7	38.9
**Occupation** *	Environmental, and occupational health and hygiene professionals	4	22.2
	Environmental, and occupational health inspectors and associates	2	11.1
	Human resources manager	1	5.6
	Human resources officer	3	16.7
	Policy officer	1	5.6
	Specialist nurse	2	11.1
	Health professionals	2	11.1
	Production and specialized services managers	1	5.6
	Teaching professionals	1	5.6
	Information and communications technology professionals	1	5.6
**Sector^‡^**	Manufacturing	6	33.3
	Electricity, gas, steam and air conditioning supply	1	5.6
	Transportation and storage	2	11.1
	Renting, buying and selling of real estate	1	5.6
	Education	1	5.6
	Human health and social work activities	7	38.9
**Number of employees**	0–50	5	27.8
	51–100	1	5.6
	101–500	1	5.6
	501–1000	2	11.1
	1001–2000	6	33.3
	>2000	3	16.7
		*Mean*	*SD*
**Self-efficacy** (N=16)	Before training	3.5	0.64
**Self-efficacy** (N=17)	After training	3.6	0.88
**Intention** (N=17)	After training	3.8	0.83

† Low: none completed, primary school and lower secondary; Moderate: middle secondary; High: upper secondary and university.

* International Standard Classification of Occupations 2008 (ISCO 2008).

‡ Standard Industrial Classification of All Economic Activities (ISIC).

#### Reasons to participate in the communication training

The interviewees provided various reasons for their participation in the communication training. Many expressed their desire to acquire practical knowledge and tools for implementing smoking cessation interventions within their companies in the future. One participant stated:

*‘We recognize that smoking causes a lot of health issues. Within our organization, we focus on sustainable employability and smoking is a very big part in that. So that is why we thought we should participate, we should gather information and see which tools we would be able to offer. How could we support our employees?’* (Participant 16, man, small company)

The interviewees emphasized one of the most important reasons for their participation, which was to obtain information on effectively communicating with and motivating employees to engage in smoking cessation interventions, which was the communication training’s primary objective:

*‘I think really everyone knows these days that smoking is not healthy and yet it remains difficult within your organization to call people on it. How do you do that?’* (Participant 11, woman, small company)

Additionally, participants expressed their desire to learn about new strategies and experiences from other organizations. They also sought guidance on how to handle negative reactions from people who smoke when addressing smoking behavior and gain insights into people’s perspectives on smoking cessation interventions. One participant expressed:

*‘We want to train supervisors and see how you approach employees that are fiercely opposed.’* (Participant 9, woman, large company)

#### Lessons learned from the communication training

All participants highly valued the communication training, although their responses varied regarding the most valuable aspect. The majority mentioned that they gained the most knowledge about practical approaches to addressing smoking in the workplace. As the communication training primarily focused on personal communication, participants learned the importance of personally engaging with employees and initiating conversations without judgment. One participant expressed their realization, stating:

*‘The eye-opener was: If you want to see effect, you have to have one-to-one conversations. We now communicate on paper, or via screens. But really approaching people in a personal way, we haven't done yet. I think that will really show more effects than the methods we used before’* (Participant 8, man, large company)

On the one hand, the role-play convinced some participants that talking about smoking with employees is acceptable:

*‘I found it a bit difficult to personally address people on that topic [smoking]. And from that training, I thought: no, it's not strange at all, if you do it in the right way, you can really talk to people about it.’* (Participant 11, woman, small company)

On the other hand, the training also made some participants think that talking about smoking with employees may require some effort, including personal, repeated contact:

*‘And I also realized that you can't just approach someone out of the blue; that doesn't work. That person thinks: “yeah, who are you? What’s your business with this?”. So you have to build or already have a relationship, and that’s maybe how you can eventually make it work. And even then, it’s step by step; it’s not like you can just tell someone, “Well, now it’s time [to quit smoking]”.’* (Participant 7, man, large company)

Furthermore, participants understood from the training that smoking is an addiction that is challenging for people who smoke heavily to overcome:

*‘Smoking is truly an addiction, so you must realize very well that people often do want to get rid of it, but it’s just really difficult to follow through and get through it.’* (Participant 7, man, large company)

Additionally, participants learned about relevant facts and figures about smoking, the role of managers in motivating employees, the benefits of using peers as ambassadors to promote the smoking cessation training, and the experiences of other companies in achieving a smoke-free environment.

#### Application of personal communication

After the training, most participants had indicated that they were committed to personally address employees who smoke about quitting in the next three months (Table 1). However, among the participants that were interviewed, in the one to five months between the communication training and the interview, only five out of eighteen participants had talked on a personal level with employees about smoking. Some participants had experience with addressing smoking behavior in the workplace previous to the communication training, while others mentioned that they had not done it and were hesitant to initiate such conversations themselves:

*‘It is not something I would easily start doing myself.’* (Participant 18, woman, large company)

Some participants were more enthusiastic about using ambassadors to communicate about smoking behavior:

*‘I think it is motivating for smokers when they hear about other’s success stories’* (Participant 10, woman, large company)

Others mentioned utilizing (digital) newsletters to reach their employees, despite the fact that they learned in the training that this is not effective.

#### Barriers and facilitators to personal communication

##### Personal barriers and facilitators

Despite acquiring knowledge on conducting effective conversations about smoking during the communication training, numerous participants still experienced a lack of confidence when it came to addressing smoking behavior in the workplace (Table 2). One participant expressed their insecurity, stating:

*‘I am a still a bit too insecure about whether colleagues are open to these conversations with me’* (Participant 12, woman, medium company)

**Table 2:** Barriers and facilitators to personal communication on the personal, interpersonal and organizational level

*Barriers*	*Facilitators*
**Personal level**	
Lack of confidence in communication skills	Strong belief in one’s own ability to succeed
Personal communication requires a significant time investment	Being able to practice communication skills
Limited motivation or willingness to take action	
It does not align with their job description	
**Interpersonal level**	
Dealing with negative reactions from people who smoke based on past experiences	Favorable encounters in dealing with smoking behavior
Discussing smoking behavior is considered a socially sensitive topic	
**Organisational level**	
The company does not provide a dedicated smoking cessation program	When the company has smoke-free regulations
Clients/patients engage in smoking	When national laws mandate companies to implement smoke-free policies
Working remotely or not being physically present in the workplace	Assistance provided by colleagues or departments within the organization
Lack of support from within the organization	Examples of successful outcomes from other organizations

The belief that colleagues were unwilling to discuss their smoking behavior negatively impacted participants’ self-efficacy. One manager stated:

*‘I will not actively address people’s smoking behavior. They will not be pleased with me.’* (Participant 10, woman, large company)

Respondents felt that there was a taboo surrounding addressing personal choices related to smoking behavior in the workplace. One manager expressed:

*‘People have a free choice in deciding to quit smoking or not. As a company, you do not want to paternalize or force your employees on such personal aspects.’* (Participant 7, man, large company)

Some participants expressed that practicing a conversation about smoking would help improve their communication skills, which was not part of the webinar:

*‘Ultimately, of course, you learn the most by just practicing a lot yourself, and a webinar is not a good method for that.’* (Participant 9, woman, large company)

*‘Maybe you could give some kind of workshop of how you learn that communication. Yes, that you role-play yourself, that you learn that communication skill a little bit.’* (Participant 8, man, large company)

Despite the participants’ limited belief in their ability to successfully address their colleagues’ smoking behavior, the majority strongly believed in the positive outcomes of personal communication. However, many participants emphasized the importance of intrinsic motivation of the employee. One manager remarked:

*‘If you smoke and you don't want to quit, you won't. Then you can offer hundreds of programs, workshops, trainings and coaches. But if you don't want to quit, you won't.’* (Participant 10, woman, large company)

Yet, some participants expressed a more nuanced view on active and personal communication, acknowledging its benefits but recognizing the challenges. One manager stated:

*‘I think the personal aspect is positive, but not easy. It remains a “taboo-topic” and I don’t think that employers could easily apply this personal approach. But I still think it is the best method.’* (Participant 15, man, medium company)

Regardless of positive attitudes towards personal communication, the intention to implement it varied. Participants perceived the personal approach to communicating about smoking cessation as time-consuming. Combined with the perception that smoking cessation was not a priority, many participants remained hesitant. They pointed to the increased workload due to COVID-19, stating:

*‘With Corona and all kinds of other business processes that take a lot of time, it is not a priority.’* (Participant 17, man, small company)

##### Interpersonal barriers and facilitators

Anticipated negative reactions or negative past experiences with co-workers were reported by some respondents as barriers to addressing smoking cessation. One participant who works in a revalidation hospital said:

*‘I thought: I really know now how to steer that conversation in the right direction. And the very first revalidation patient I approached, well, he opened his mouth, he wanted to sue me.’* (Participant 2, woman, large company)

However, participants believed that addressing smoking cessation would be facilitated if it aligned with their job description. Many participants mentioned that addressing employees personally was not part of their job, and therefore, they did not employ personal communication strategies during work hours.

Despite gaining knowledge from the communication training, many participants did not feel responsible for sharing the recording of the communication training with their colleagues, as they believed their colleagues would not watch it anyway. One manager expressed:

*‘It [the recording] is very easy to share, but it [smoking cessation] just does not speak to people. We are busy with, what I notice, absenteeism, holding the team together, COVID vaccinations, and hoping that we get through this winter season steadily. And corona, digitalization, working from home.’* (Participant 4, woman, large company)

##### Barriers and facilitators related to the organization

While participants generally expressed positivity towards personal and active communication, many expressed concerns about the supportiveness of their supervisors in offering smoking cessation programs. They observed a lack of support from within their companies, with smoking often not being considered a priority, despite its recognized importance.

Participants mentioned that communication about smoking cessation at work becomes easier when there is a specific smoking policy in place. In addition, participants noted that it was important to have support within the organization:

Participant 6 (woman, medium company): *‘I'm convinced that we could do something for our people [to stimulate smoking cessation] and that we should. But if I'm the only one standing up for it, that makes things quite difficult. Now, we are in the process of hiring a new colleague, a Sustainable Employability Advisor, and I honestly expect that this will be one of the topics they will need to focus on.’*

Interviewer: *‘So, this new colleague could actually help you within the organization to develop policies around this and also support you so that you don’t feel like you’re alone in it?’*

Participant 6: *‘Yes, that we're in it together and that we can take it on and make it happen as a team.’*

In addition to a smoking policy at the workplace, many recognized the significance of a national antismoking policy or law in facilitating discussions about smoking cessation. When companies are mandated by law to become smoke-free, participants cannot be seen as the sole responsible parties:

*‘That they for example say: “ from the 1st of January 2023, you can't smoke at the worksite anymore”. Then you at least have some sort of law, just like with these indoor smoking areas that were banned.’* (Participant 10, woman, large company)

Lastly, participants acknowledged that having individual conversations with colleagues and employees became challenging due to remote work measures implemented by their companies. Working from home posed difficulties for participants in having face-to-face discussions about smoking:

*‘I work a lot remote now due to the COVID restrictions, so it is difficult to plan.’* (Participant 6, woman, medium company)

### One year after the communication training

One year later, all participants still maintained a positive attitude towards personal communication. However, only one manager implemented the personal communication strategy at work and had engaged in conversations with 5 to 10 employees since the initial interview. Most others had not or only incidentally personally communicated with employees. In practice, the participants had not found a way to overcome the barriers hindering personal communication. Many participants emphasized that engaging in personal communication did not align with their job description and, therefore, they did not feel responsible for addressing smoking behavior in the workplace. As one manager stated:

*‘I'm not the person to address people for that. (...) that conversation really lies in the relationship of trust with managers, the team coordinator.’* (Participant 2, woman, large company)

Moreover, only a few companies had made changes to their smoking policies or had provided smoking cessation support, and in the absence of a specific smoking cessation policy or training, participants remained hesitant to address smoking cessation. Furthermore, participants expressed that they had other priorities and, therefore, had not yet addressed smoking within their companies, although they had it on their agenda for future change:

*‘I think you first have to make it clear in your communication: where do we stand with our main policy and where are we going?’* (Participant 8, man, large company)

In conclusion, one year after the training, personal communication with employees about smoking was not successfully implemented or maintained in most organizations due to perceived role boundaries, lack of organizational support or policy, and competing priorities.

## DISCUSSION

This study aimed to evaluate a communication training designed to encourage managers to engage in personal communication with employees about workplace smoking cessation programs. The findings revealed that among those who completed the questionnaires and interviews, the training increased participants’ understanding of the importance of personal communication in promoting smoking cessation among employees, and fostered a positive attitude and intention to apply this approach in practice. Despite this, few participants had actually personally communicated about smoking cessation with their employees or implemented this communication strategy within their organization. The interviews revealed several barriers and facilitators encountered by managers in implementing personal communication strategies and addressing smoking behavior in the workplace.

Although participants had rated their selfefficacy for personally communicating as fairly high on the questionnaires, the interviews painted a more nuanced image. Many interviewees still lacked confidence to address smoking behavior. Managers were concerned about potential negative reactions from employees and expressed the belief that smoking may be considered a personal choice and that discussing this behavior could be seen as intrusive and paternalistic. These concerns negatively affected their willingness to engage in personal conversations with employees about smoking. Another major barrier was that while managers recognized that a personal and proactive approach would yield the best results in reaching their employees, many did not feel responsible for undertaking this task. Our findings align with existing literature, indicating that managers are uncertain about their responsibility to offer healthpromoting interventions in the workplace, despite understanding the potential benefits. Research by McCoy et al.^22^ demonstrated that employers in small businesses were reluctant to intervene in their employees’ personal lifestyle choices. Similarly, related literature shows that managers are hesitant to address health promotion activities at work due to concerns about intruding into their employees’ personal lives and perceiving it as outside their job role^15,23,24^. Instead, employers often delegate this task to colleagues who are more directly involved, such as ambassadors or people who used to smoke. Finally, important barriers related to the organization were revealed. A lack of prioritization of smoking cessation was considered a barrier, while clear smoking cessation company policies and the availability of a smoking cessation support program for employees were considered facilitators for engaging in conversations about smoking with employees.

The results from this study show that to enhance the effectiveness of workplace smoking cessation programs, organizations will need to employ strategies to address the identified barriers. It is evident that this single training in the form of a communication training was insufficient to stimulate managers to implement proactive and personal communication about smoking with their employees. Managers with low self-efficacy and skills in having conversations about smoking, may need additional training, for example an in-person training with multiple sessions, allowing them to practice their conversation skills through role-plays and build confidence in addressing smoking behavior^25^. If managers actually try out talking about smoking with an employee, and gain positive experiences from these conversations, their beliefs about negatively intruding in an employee’s personal life may be positively changed resulting in applying this communication strategy more often.

If organizations want to effectively stimulate smoking cessation among employees with a lower socioeconomic position, they need to prioritize smoking cessation programs, establish clear smoking policies, and create a supportive environment that encourages managers to engage in personal communication. Organizations should always have an offer for a smoking cessation program that managers could refer to employees who need support with quitting smoking, or organizations would need to keep their managers informed about what type of programs are available in standard health care. Sustained implementation may thus depend on embedding the training within a broader organizational health program. One example of a practical template for organizations to build a workplace health program is the CDC Workplace Health Model, which offers an iterative four-step cycle of assessment, planning, implementation, and evaluation, supported by strong leadership commitment, a designated program coordinator, and continuous quality improvement^26^. Applying such a framework can help organizations to translate a single training session into measurable objectives, such as the proportion of supervisors who initiate personal cessation conversations, allocate the necessary resources, and monitor progress over time, ensuring that smoking-cessation support becomes an integral, enduring component of organizational practice.

### Limitations

It is important to acknowledge the limitations of this study. Our expert panel that co-developed the training was entirely composed of individuals born and educated in the Netherlands, which may limit the study’s generalizability. Furthermore, only about half of the communication training participants completed the post-communication training survey, and for the interviews, we approached only those participants who expressed willingness to participate, which may introduce selection bias. Additionally, managers who had already implemented strategies from the communication training were more willing to participate in the second interview, while organizations that had not yet initiated smoking cessation activities or intended to do so in the future were less inclined to participate.

## CONCLUSIONS

While participants expressed a positive attitude regarding proactive and personal communication strategies to address smoking and were positive about the offered training in which they learned this, the actual implementation remained limited. A more comprehensive training, offering greater opportunities to practice conversations and explore various scenarios may enhance managers’ confidence in their communication skills. In addition, a supportive organizational environment that prioritizes smoking cessation programs is necessary to increase managers’ willingness to address smoking behavior with employees.

## Supplementary Material



## Data Availability

The data supporting this research are available from the authors on reasonable request. Some of the materials can be accessed via www.samensterkerstoppen.nl.
