# Supplementary material for: Evaluation of a training for managers in using active and personal communication to stimulate employee participation in a workplace smoking cessation program
Source: Tob Prev Cessat. 2025 Aug 29;11:10.18332/tpc/208807. doi: 10.18332/tpc/208807 (PMC12395514; doi:10.18332/tpc/208807)
Supplement: Supplementary file 1 [file TPC-11-41-s1.pdf]

**SUPPLEMENTARY FILE**

## Material 1: Interview guide Round 1

### Employer Background

- What is your age, education, and job title?
- What type of company do you work for? In which industry?
- What is the background of your company's employees? (Number of employees, education level, number of employees with low socioeconomic status, smoking behavior, etc.)
- Does your company have a policy to encourage smoking cessation? If so, what does it include?
- Does your company offer smoking cessation support?

### Introductory Questions

- How did you hear about the webinar?
- Did you attend the webinar live or watch it online afterward?
    - If you watched it online: Did you watch the entire webinar or just specific video clips? Which clips did you watch?
- Why did you participate in the webinar/watch it online?
- What was the most useful part of the webinar for you? What specifically did you learn?
- What was the least useful part?

### Practical Application & Barriers

- Have you communicated with employees about smoking cessation since the webinar?

**If YES:**

- Were you already doing this before the webinar? If so, what was different this time?
- What communication methods did you use?
- Can you tell me more about your communication with employees?
- How many employees did you talk to? Did you set a goal for the number of employees you wanted to reach?
- How did these conversations go?
- What were the results of these conversations? Did they differ from previous experiences? Did you follow up later?
- To what extent did the webinar help you initiate and conduct these conversations? What specifically helped you?

- What barriers still exist for you in communicating personally with employees about smoking cessation? What would help you overcome these barriers?

**If NO:**

- Do you plan to communicate personally with employees about smoking cessation in the near future? If so, when?
- Why haven't you (yet) spoken with employees about smoking cessation?
- What would help convince you or remove these barriers?
- What could we have done in the webinar to convince you or help remove these barriers?
- What could we have sent you after the webinar to convince you or help remove these barriers?
- What could we include in a follow-up webinar or other sessions to convince you or help remove these barriers?

**Colleague Engagement**

- Following the webinar, have you encouraged colleagues to engage in personal communication?
- Have you shared (the information from) the webinar within your organization?

**Impact of the Webinar on Key Determinants**

- What do you think about personal communication with employees about smoking cessation? Do you see it as positive or negative, and why? To what extent has the webinar influenced your opinion?
- To what extent do you believe personal communication with employees can help encourage smoking cessation within your organization? To what extent has the webinar influenced your opinion?
- Has the webinar increased your confidence in personally communicating with employees about smoking cessation?

**Website**

- Have you visited the *Samen Sterker Stoppen* website since the webinar?
- What did you look at, and what did you think of it?

**Material 2: Interview guide Round 2**

**Employer Background**

- Are you still working at the same organization? Has your role changed?
- Does your company have a policy to encourage smoking cessation? If so, what does it entail?
- Has your organization offered any smoking cessation support to employees in the past year (since the last interview)? If so, what exactly?
- Is there a plan to offer smoking cessation support in the future?

**Website Review**

- Have you visited the *Samen Sterker Stoppen* website in the past year? (Check if this was only due to the interview request.)
- If yes: What did you look at, and what did you think of it? Did you do anything with the information?
- If no: Why haven't you visited the website?
- Have you looked at other websites with information on smoking cessation? Which ones?

**Practical Application & Barriers**

- Since the last interview, have you communicated with employees about smoking cessation?

**If YES:**

- What communication methods did you use?
- Can you tell me more about your communication with employees?
- How many employees did you speak with? Did you set a goal for the number of employees you wanted to reach?
- How did these conversations go?
- What was the outcome of these conversations? Did you follow up later?
- To what extent did the webinar help you initiate and conduct these conversations? What specifically helped you?
- Do you plan to continue personal communication with employees about smoking cessation?
- What barriers do you still face in communicating personally with employees about smoking cessation? What would help you overcome these barriers?

**If NO:**

- Do you plan to start personal communication with employees about smoking cessation? If so, when?
- Why haven't you (yet) spoken with employees about smoking cessation?

- What would help convince you or remove these barriers?
- What could we include in a follow-up webinar or other sessions to help convince you or address these barriers?

**Colleague Engagement**

- Have you encouraged colleagues to engage in personal communication over the past year?
- Have you shared (the information from) the webinar or website within your organization?
- Have your colleagues had conversations with employees about smoking cessation?
- What would be needed within your organization to encourage personal communication with employees about quitting smoking?

**Impact of the Webinar on Key Determinants**

- What do you think about personal communication with employees about smoking cessation? Do you see it as positive or negative, and why?
    - Has your opinion changed in the past year (since the last interview)? If so, why?
- To what extent do you believe personal communication with employees can contribute to promoting smoking cessation within your organization?
    - Has your opinion on this changed in the past year? If so, why?
- How confident do you feel in having personal conversations with employees about smoking cessation?
    - Has your confidence changed in the past year? If so, why?

**Material 3: Overview of participants' learning objectives, as indicated in the registration forms, summarized and structured by thematic categories**

| Theme and main questions | Included in training |
|---|---|
| **Starting the conversation**<br>• How to talk about quitting smoking in a respectful, non-judgmental way.<br>• Who is best suited to start the conversation (e.g. HR, manager).<br>• How to tailor the message depending on the employee's attitude and readiness. | Yes |
| **Motivating employees**<br>• How to encourage employees who are not intrinsically motivated.<br>• What arguments or benefits (e.g. health, finances, social) work best to engage employees who smoke.<br>• How to inspire people to attend quit-smoking sessions or use available support. | Yes |
| **Creating a supportive culture**<br>• How to build a culture where smoke-free living is the norm.<br>• How colleagues can speak to each other about smoking in a constructive way.<br>• How to shift from general policies to more personal, employee-focused approaches. | Partly |
| **Dealing with resistance and relapse**<br>• How to handle resistance to employer-organized training.<br>• How to support employees during and after quitting, especially around relapse risks.<br>• How to respond to common objections ("I'd rather live shorter", "It's my choice", etc.). | Yes |
| **Practical tools and examples**<br>• Examples from other organizations about what works.<br>• Effective communication strategies and rewards that don't create jealousy.<br>• Tips for long-term support, monitoring, and motivation. | Yes |
| **Implementing smoke-free policies**<br>• Practical steps to make the workplace or site smoke-free.<br>• Addressing common barriers like high smoking prevalence or logistical issues.<br>• Balancing health goals with continuity of care (e.g. in healthcare settings).<br><br>*Not the aim of the training, but referral to relevant sources was included. | No* |

© 2025 van den Brand F.A. et al.
